# Heterologous boosting with third dose of coronavirus disease recombinant subunit vaccine increases neutralizing antibodies and T cell immunity against different severe acute respiratory syndrome coronavirus 2 variants

**DOI:** 10.1080/22221751.2022.2048969

**Published:** 2022-03-15

**Authors:** Zhuxiang Zhao, Tingting Cui, Mingzhu Huang, Shuo Liu, Xiaoling Su, Guichang Li, Tao Song, Weidong Li, Nanshan Zhong, Miao Xu, Xiaoyun Yang, WeiJin Huang, Zhongfang Wang

**Affiliations:** a Department of Infectious Disease, Respiratory and Critical Care Medicine, Guangzhou First People’s Hospital, Guangzhou Medical University, Guangzhou, People’s Republic of China; b State Key Laboratory of Respiratory Disease & National Clinical Research Center for Respiratory Disease, Guangzhou Institute of Respiratory Health, the First Affiliated Hospital of Guangzhou Medical University, Guangzhou Medical University, Guangzhou, People’s Republic of China; c Guangzhou Laboratory, Bioland, Guangzhou, People’s Republic of China; d National Institutes for Food and Drug Control, NHC Key Laboratory of Research on Quality and Standardization of Biotech Products & NMPA Key Laboratory for Quality Research and Evaluation of Biological Products Beijing, People’s Republic of China

**Keywords:** COVID-19 vaccine, prime-boost strategy, neutralizing antibody, T cell response, heterologous boosting, subunit vaccine, inactivated vaccine

## Abstract

Waned vaccine-induced immunity and emerging severe acute respiratory syndrome coronavirus 2 variants with potential for immune escape pose a major threat to the coronavirus disease (COVID-19) pandemic. Here, we showed that humoral immunity components, including anti-S + N, anti-RBD IgG, and neutralizing antibodies (NAbs), gradually waned and decreased the neutralizing capacity against emerging Omicron variants at 3 and 6 months after two inactivated COVID-19 vaccinations. We evaluated two boosting strategies with either a third dose of inactivated vaccine (homologous, I-I-I) or a recombinant subunit vaccine (heterologous, I-I-S). Both strategies induced the production of high levels of NAbs with a broad neutralizing capacity and longer retention. Interestingly, I-I-S induced 3.5-fold to 6.8-fold higher NAb titres than I-I-I, with a broader neutralizing capacity against six variants of concern, including Omicron. Further immunological analysis revealed that the two immunization strategies differ considerably, not only in the magnitude of total NAbs produced, but also in the composite pattern of NAbs and the population of virus-specific CD4+ T cells produced. Additionally, in some cases, heterologous boosted immunity induced the production of more effective epitopes than natural infection. The level of I-I-S-induced NAbs decreased to 48% and 18% at 1 and 3 months after booster vaccination, respectively. Overall, our data provide important evidence for vaccination strategies based on available vaccines and may help guide future global vaccination plans.

## Introduction

Severe acute respiratory syndrome coronavirus 2 (SARS-CoV-2) has caused more than 300 million infections and 5.46 million deaths, with major losses to human health and economies worldwide. Vaccination is one of the safest methods to control the pandemic and restore global health. However, the duration of vaccine-induced immunity is a major concern. Several studies have indicated that vaccination can only confer 6–8 months of protective immunity against severe disease and death [1,2]. Therefore, measures to induce higher levels of neutralizing antibodies (NAbs) and T-cell immunity are of great significance. Additional efforts, including strategies for antigen optimization, vaccine development, and adjuvant selection, are necessary to achieve this goal. In recent years, some researchers have experimented with heterologous prime-boost (mix-match) by switching from one vaccine to another for the second dose, which provides promising protection efficiency [3–6]. However, there remains a long-running debate on whether a mix-and-match strategy helps protect individuals from SARS-CoV-2. Reportedly, boosting with a recombinant subunit, adenoviral, or mRNA vaccine after two doses of inactivated vaccine could improve NAb titres in a mouse model [7]. Several studies have shown that the administration of an mRNA vaccine followed by an adenovirus vector-based coronavirus disease (COVID-19) vaccine induced a strong immunogenic response, with the production of high levels of NAbs and a strong T cell response [8–10], which indicates the potential for the strategic optimization of vaccine efficacy.

Meanwhile, the frequent emergence of new variants, such as Delta and Omicron, increases the chances of the virus escaping from population immunity induced by natural infection or vaccination. A recent report showed that the Omicron strain could decrease the mRNA vaccination-induced neutralizing capacity by 43-fold, which would help almost completely overwhelm the vaccine-induced protective immunity, even in very early phases of vaccination [11]. This will reduce the protective window conferred by vaccines. Therefore, new vaccines or immune strategies are needed for the development of stronger protective immunity against emerging variants, such as Omicron.

Here, we assessed a vaccination strategy with two shots of inactivated vaccine followed by a third shot of a recombinant subunit vaccine (heterologous, I-I-S), as compared to three shots of inactivated vaccine administered to the control group (homologous, I-I-I). The heterologous booster induced higher levels of NAbs and stronger RBD-specific CD4+ T cell immunity than homologous enhancement vaccination, and also induced the production of NAbs against six other variants of concern (VOCs), including Omicron, at considerable levels. Overall, our findings highlight the importance of heterologous vaccination and provide guidance for new vaccination strategies.

## Materials and methods

### Study design and participants

This study was designed to emulate a target trial on the effect of a third dose of RBD recombinant subunit vaccine (Zifivax) (I-I-S) and an inactivated vaccine (CoronaVac or BBIBP-CorV) (I-I-I) in a population previously administered two doses of inactivated vaccine (CoronaVac or BBIBP-CorV) at least 3 months (3M) before recruitment. This study comprised a small group of volunteers who know the aim of the study and signed informed consents. The I-I-I group comprised 38 participants with a median age of 43 years (interquartile range (IQR), 37–50 years). The I-I-S group comprised 27 participants with a median age of 28 years (IQR, 25–30 years). Thirty-one (81.6%) participants in the I-I-I group and 21 (77.8%) participants in the I-I-S group were women.

This study was approved and supervised by the GMUH Ethics Committee (No.2021-78).

### iFlash-SARS-CoV-2 IgG assay

The iFlash-SARS-CoV-2 IgG assay is a paramagnetic particle chemiluminescent immunoassay for the qualitative detection of anti-S + N IgG antibodies against SARS-CoV-2 in human sera or plasma using the iFlash Immunoassay Analyzer. The assay was performed in accordance with the manufacturer’s instructions (Shenzhen Yhlo Biotech, Shenzhen, China). Plasma samples collected from heparinized whole blood by centrifugation at 800 g for 10 min were first incubated with SARS-CoV-2 antigen-coated paramagnetic microparticles to allow the binding of antibodies to the coated antigen. An acridinium-labelled anti-human IgG conjugate was then added to form a reaction complex. Following the addition of trigger solutions to the reaction mixture, the anti-S + N IgG titre was measured using an iFlash optical system.

### Enzyme-linked immunosorbent assay (ELISA)

To detect specific plasma anti-RBD IgGs in the vaccination samples which were collected from heparinized whole blood by centrifugation at 800 g for 10 min, a direct ELISA was conducted against the SARS-CoV-2 S protein RBD domain using a commercial ELISA kit (Darui Biotechnology, Guangzhou, China). A sample with a high anti-RBD titre was used as the standard sample, with an arbitrary unit (unit/mL) of 500. Next, a 2-fold serial dilution of the standard sample was performed at each instance for the evaluation of other plasma samples according to the manufacturer’s instructions. A washing buffer was used as the blank control. Absorbance was measured at 450 nM by a Multiskan GO microplate spectrophotometer (Thermo Fisher Scientific, Waltham, MA, USA). Data were analysed using a standard curve with a log-logistic model.

### NAb test based on ace2 competitive ELISA

ACE2 competitive ELISA for NAb testing was conducted to mimic SARS-CoV-2 infection. A sample with a high NAb titre was used as the standard with an arbitrary unit (unit/mL) of 1000. A standard curve was obtained with 2-fold serial dilution according to the protocol provided by the manufacturer. Standards and plasma samples were pre-treated with a horseradish peroxidase (HRP)-labelled RBD antigen. Negative serum was used as the control. A mixture of plasma and antigen was added to the ACE2-coated plate. After incubation with the substrate solution, absorbance was measured at 450 nM using a Multiskan GO microplate spectrophotometer (Thermo Fisher Scientific, Waltham, MA, USA). Thereafter, data were analysed using a standard curve with a log-logistic model.

### SARS-CoV-2 pseudovirus-based neutralization test

Wuhan-1 and −6 variants were examined as representatives of the original SARS-CoV-2 strain and emerging variants with mutations in the spike protein, respectively. Neutralization was measured by suppressing Luc gene expression, as previously described for the HIV pseudovirus neutralization assay [12]. The 50% inhibitory dilution (EC_50_) was defined as the serum dilution at which the relative light units (RLUs) were reduced by 50% compared to that in the virus control wells (virus + wells) after subtraction of the background RLUs in the control groups containing only cells. Briefly, the pseudovirus was incubated with serial dilutions of the test samples (six dilutions in a 3-fold stepwise manner) in duplicate for 1 h at 37 °C, along with the virus control and cell control wells in hexaplicate. Freshly trypsinized cells were added to each well. Following 24 h of incubation, luminescence was measured as described in the section on pseudovirus titrations. EC_50_ values were calculated using non-linear regression, i.e. log (inhibitor) vs. response (four parameters), using GraphPad Prism 8 (GraphPad Software, San Diego, CA, USA).

### SARS-CoV-2 conventional virus neutralization test

Plasma neutralization activity was evaluated using a CPE-based assay, as previously described [13]. Plasma samples were tested at an initial dilution of 1:8, and then diluted in steps of 1:2 for eight points. All samples were mixed with a SARS-CoV-2 Wuhan-1 Omicron viral solution containing 100 TCID_50_ of the virus. After 2 h of incubation at 37°C and 5% CO_2_, the virus-plasma mixture was added to the wells of a 96-well plate containing 1.2 × 10^4^ Vero E6 cells. Plates were incubated for 4 days at 37 °C in a humidified environment with 5% CO_2_ and then examined for CPE using a Celigo Imaging Cytometer (Nexcelom Bioscience, Lawrence, MA, USA). The absence or presence of CPE was defined by comparing each well with the wells corresponding to a positive control (plasma sample showing high SARS-CoV-2 neutralizing activity in infected Vero E6 cells) and negative control (human serum sample negative for SARS-CoV-2 in ELISA and neutralization assay and Vero E6 cells alone).

### Cell-based competitive ELISA (ccELISA) for the detection of NAb clusters

Mouse NAb clones 13G2 and 08B3 were purified using a HiTrap Protein G column (GE Healthcare Life Sciences, USA), followed by conjugation with HRP using EZ-Link Plus Activated Peroxidase (Thermo Scientific, USA) according to the manufacturer’s instructions. Cluster-targeting NAbs were measured using an in-house ccELISA. Briefly, A549 human lung carcinoma cells from ATCC were infected with Lenti-GFP-S (6P) for 48 h, and cells with high GFP expression were sorted by FACS using BD AriaIII and cultured in media supplemented with 0.5 μg/mL puromycin for 15 days. A549 cells stably expressing SARS-CoV-2 were established. The cells were seeded in 96-well plates at 2 × 10^4^ cells/well. At 24 h after seeding, the cells were fixed with 4% paraformaldehyde at room temperature for 20 min, followed by blocking for 2 h with PBST containing 3% BSA at 37°C. Next, 50 μL of each diluted serum sample was mixed with 50 μL of diluted HRP-13G2 or HRP-08B3, added to the A549 cells, and incubated at 37°C for 1 h. After extensive washing, 100 μL of TMB stabilized chromogen (Invitrogen, USA) was added, and the cells were incubated at 37 °C for 10 min, followed by treatment with 50 μL of stopping solution (R&D Systems, USA). Absorbance was measured at 450 nm using a Multiskan GO microplate spectrophotometer (Thermo Fisher Scientific, Waltham, MA, USA).

Furthermore, to standardize the assay and quantify the results, an arbitrary titre was applied to each tested sample based on the standard curve drawn from a dilution series of sera with known competition rates against 13G2 and 08B3 clones.

### Peripheral blood mononuclear cell (PBMC) isolation and ex vivo stimulation

PBMCs were isolated from heparinized whole blood by density-gradient sedimentation using Ficoll-Paque according to the manufacturer’s instructions (GE Healthcare, 17-1440-02). PBMCs (5 × 10^5^) were cultured in RPMI 1640 medium (Gibco, Waltham, MA, USA) supplemented with 10% heat-inactivated FBS (Biological Industries, Israel Beit-Haemek), 100 U/mL penicillin (Gibco, Waltham, MA, USA), and 0.1 mg/mL streptomycin (Gibco, Waltham, MA, USA). PBMCs were treated overnight using a peptide pool containing 56 15-mer peptides (2 μM per peptide) in the presence of 10 U/mL rIL-2 and 1 µM GolgiPlug (BD Biosciences, San Diego, CA, USA) at 37 °C and 5% CO_2_.

### Flow cytometry

Cells harvested from overnight stimulation cultures were incubated with live/dead aqua V510 for 15 min on ice. The cells were then surface stained for 30 min on ice using anti-CD3-FITC (BioLegend, clone UCHT1, 1:200, Cat# 300406), anti-CD4-APC-H7 (BD Pharmingen™, clone RPA-T4, 1:200, Cat# 560158), and anti-CD8-PerCPCy5.5 (BD Biosciences, clone RPA-T8, 1:200, Cat# 560662) antibodies. After fixation and permeabilization with Cytofix and Perm (BD Biosciences, Cat# 554714) on ice for 15 min, intracellular staining was performed on ice for 30 min using anti-TFNα-PE-Cy7 (BD, clone MAb11, 1:200, Cat # 557647) and anti-IFNγ-APC (BD Pharmingen™, clone B27, 1:200, Cat# 554702) antibodies. After the final wash step, the cells were resuspended in 200 µL of FACS buffer. Samples were acquired using an FACSAria III instrument (BD Biosciences, San Diego, CA, USA) and analysed using the FlowJo software (Treestar, San Carlos, CA, USA).

### Statistical analysis

We performed a paired *t*-test to analyse the antibody titres in samples collected from participants who received two doses of vaccination, which were segregated according to different time points (D7, 1M, 3M, and 6M). D0 and D14 samples from I-I-I and I-I-S groups were compared using a paired *t*-test. Antibody titres between the I-I-I and I-I-S groups were compared using an unpaired *t*-test. All analyses were performed using GraphPad Prism version 6.0 (GraphPad Software) and SPSS version 26. Differences were considered statistically significant at *p* < 0.05.

## Results

### I-I-S conferred considerably greater immunogenicity than I-I-I

**Figure 1.**
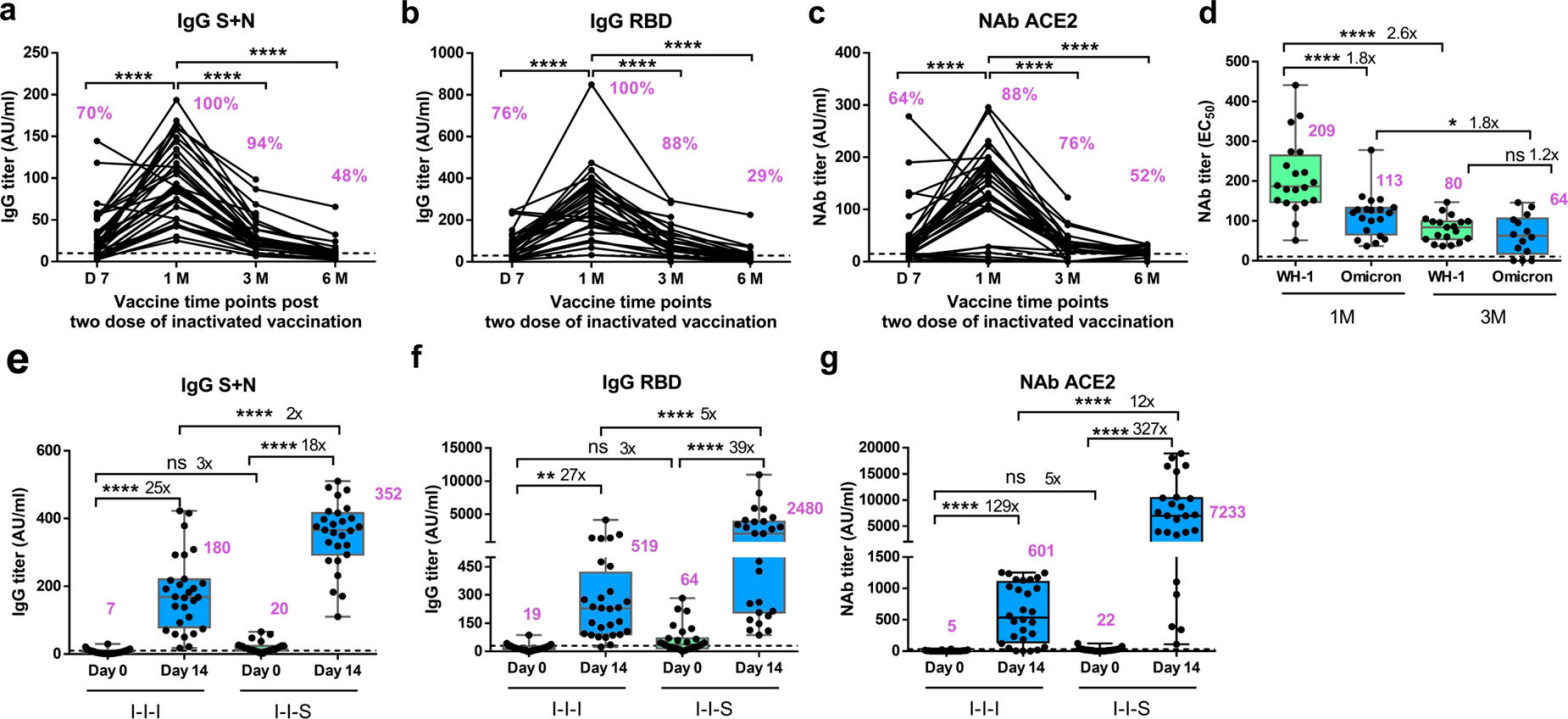
Antibody response after two-dose of inactivated vaccination and booster vaccination with RBD subunit or inactivated vaccination. (a) Total anti-S + N antibody titre of two doses of inactivated vaccines, n = 33 at D7, D14, 1, 3 M, n = 21 at 6 M. (b) Total anti-RBD antibody titre of two doses of inactivated vaccines, n = 33 at D7, D14, 1M, 3M, n = 21 at 6M. (c) Neutralizing antibody titre based on ACE2 competition assay of two doses of inactivated vaccines, n = 33 at D7, D14, 1M, 3M, n = 21 at 6M. (d) Neutralizing antibody titres against Wuhan-1 and Omicron strains for two doses of inactivated vaccines, n = 20 for Wuhan-1 and Omicron at 1M, n = 14 for Wuhan-1 and Omicron at 3M. (e) Comparison of total anti-S + N antibody titer between I-I-I and I-I-S, n = 28 in I-I-I, n = 27 in I-I-S. (f) Antibody response in booster groups evaluated by anti-RBD IgG, n = 28 in I-I-I, n = 27 in I-I-S. (g) Antibody response in booster groups evaluated by Neutralizing antibody titre based on ACE2 competition assay, n = 28 in I-I-I, n = 27 in I-I-S. ns, not significant; *P < 0.05, **P < 0.01, ****P < 0.0001.

The total anti-S + N antibody titre, anti-RBD antibody titre, or NAb titre (based on the ACE2 competition test) peaked at 1 month (1M) after two-dose inactivated vaccination, which decreased drastically to the limit of detection at 6 months (6M) post vaccination (Figure 1(a-c)). We observed that 48% of the samples showed a substantial total anti-S + N antibody titre at 6M post vaccination (Figure 1A). Meanwhile, only 29% of the samples showed a considerable anti-RBD antibody titre at 6M post vaccination (Figure 1b). At 6M post vaccination, 58% of samples tested positive for NAbs, but at considerably low levels, based on the ACE2 competition assay (Figure 1c). Importantly, compared to that at 1M post vaccination, the NAb titre against the Wuhan-1 and Omicron strains decreased significantly at 3 months (3M) post vaccination, declining from 209 (95% CI, 165–253) for Wuhan-1 and 113 (95% CI, 88–139) for Omicron at 1M to 80 (95% CI, 65–96) for Wuhan-1 and 64 (95% CI, 35–93) for Omicron at 3M. A 2.6-fold and 1.8-fold lower EC_50_ titre was observed at 3M post vaccination for the Wuhan-1 and Omicron strains, respectively (Figure 1d).

To test whether different vaccination strategies could induce varied immune responses, we measured the anti-S + N IgG and anti-RBD IgG titres on day 0 (D0) and day 14 (D14) after the third shot of the RBD recombinant subunit vaccine (I-I-S) versus inactivated vaccine (I-I-I) preceded by two inactivated vaccines. Both vaccines induced significantly higher levels of anti-S + N IgG and anti-RBD IgG on D14 than on D0. In the I-I-S group, anti-RBD IgG was present at 39-fold higher levels, and anti-S + N IgG was present at 18-fold higher levels. In the I-I-I group, the anti-RBD IgG antibody titre was 27-fold higher, and the anti-S + N IgG antibody titre was 25-fold higher (Figure 1e and f). However, I-I-S induced a 2-fold greater increase in the anti-S + N IgG titre and a 5-fold greater increase in the anti-RBD antibody titre than I-I-I.

To investigate whether the vaccine strategies could induce different levels of NAbs, we measured the NAb titres using an ACE2 competition assay at D0 and D14. The third shot of both vaccinations significantly increased the NAb titre against ACE2; the I-I-S group showed 7-fold greater NAb titres against ACE2 binding than the I-I-I group at D14 post booster vaccination (Figure 1 g).

### I-I-S conferred a stronger neutralizing capacity against other VOCs, including omicron, than I-I-I

**Figure 2.**
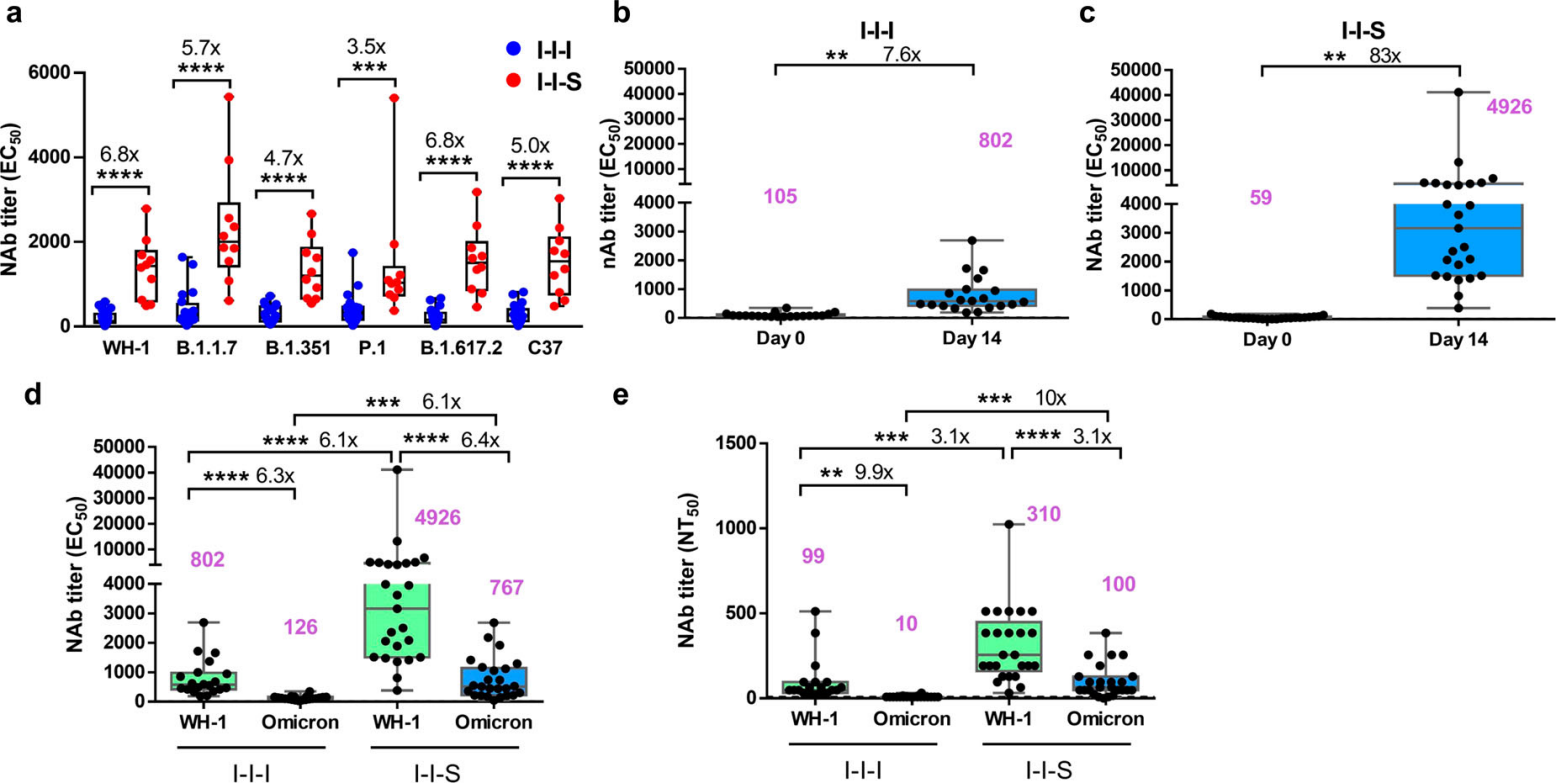
Neutralizing activity comparison between I-I-I and I-I-S. (a) Pseudovirus-based neutralizing response after two strategies of booster vaccinations against Wuhan-1 and VOCs, including, B.1.1.7 (Alpha Variant), B.1.351 (Beta Variant), P.1 (Gamma Variant), B.1.617.2 (Delta Variant) and C37 (Lambda Variant) strains. (b) EC_50_ titer after third shot of inactivated vaccine against Wuhan-1 stain (n = 20). (c) EC_50_ titer after third shot of RBD recombinant subunit vaccine against Wuhan-1 stain (n = 25). (d) Comparison of the neutralizing titer based on pVNT against Omicron strain between I-I-S (n = 25) and I-I-I (n = 20). (e) Neutralizing antibody titres against Wuhan-1 strain and Omicron strain for I-I-S (n = 25) and I-I-I (n = 20) evaluated by conventional virus neutralization test. **P < 0.01, ***P < 0.001, ****P < 0.0001.

Both boosting strategies induced high levels of NAbs against different VOCs. Table 1 shows the mean EC_50_ values for Wuhan-1 and the different VOCs. We found that I-I-I induced NAbs against B.1.1.7 at EC_50_ 412 (95% CI, 203–620), which was significantly lower than that induced by I-I-S (EC_50 _= 2341, 1333–3349). Similarly, compared to I-I-I, I-I-S induced 4.7-fold, 3.5-fold, 6.8-fold, and 5-fold higher NAb titres against the B.1.353, P.1, B.1.617.2, and C37 strains, respectively (Figure 2a).

**Table 1.** Neutralizing antibody titre against Wuhan-1 strain and different variants of concern.

		Mean	Std. Deviation	Std. Error of Mean	Lower 95% CI of mean	Upper 95% CI of mean
**I-I-I**	WH-1	201	165	37	123	278
	B.1.1.7	412	446	100	203	620
	B.1.351	284	200	45	191	377
	*P*.1	414	389	87	231	596
	B.1.617.2	229	189	42	140	317
	C37	308	223	50	203	412
**I-I-S**	WH-1	1373	725	229	854	1891
	B.1.1.7	2341	1409	446	1333	3349
	B.1.351	1344	709	224	836	1851
	*P*.1	1444	1452	459	406	2483
	B.1.617.2	1560	798	252	989	2130
	C.37	1539	805	255	963	2115

To evaluate the protective potential of booster vaccination against Omicron infection, a pseudovirus-based neutralization test against the Wuhan-1 and Omicron strains was performed using plasma collected at D0 and D14 after booster vaccination. The results showed that in the I-I-I group, the EC_50_ titre against the Wuhan-1 strain at D14 (EC_50 _= 802, 95% CI, 509–1095) was 7.6-fold higher than that at D0 (EC_50 _= 105, 95% CI, 69–140) (Figure 2b). Surprisingly, in the I-I-S group, the EC_50_ titre at D14 (EC_50 _= 4926, 95% CI, 1627–8225) was 83-fold higher than that at D0 (EC_50 _= 59, 95% CI, 40–78) (Figure 2c). Compared to the I-I-I group, the I-I-S group showed a significantly higher (6.1-fold) EC_50_ titre against the Wuhan-1 strain (Figure 2d). Meanwhile, in the I-I-I group, the EC_50_ titre against Omicron (EC_50 _= 126, 95% CI, 94–158) was 6.3-fold lower than that against Wuhan-1 (EC_50 _= 802, 95% CI, 509–1095), whereas in the I-I-S group, the titre against Omicron (EC_50 _= 767, 95% CI, 482–1051) was 6.4-fold lower than that against Wuhan-1 (EC_50 _= 4926, 95% CI, 1627–8225). Similarly, the EC_50_ titre against Omicron in the I-I-S group was significantly higher (6.1-fold) than that in the I-I-I group (Figure 2d).

Furthermore, an authentic virus neutralization test was performed to determine the neutralizing titre of plasma samples collected after booster vaccination. The neutralizing titre of I-I-S against Wuhan-1 (NT_50 _= 310, 95% CI, 221–399) was significantly higher (3.1-fold) than that of I-I-I (NT_50 _= 99, 95% CI, 39–158). In addition, the neutralizing titre of I-I-S against Omicron (NT_50 _= 100, 95% CI, 60–140) was significantly higher (10-fold) than that of I-I-I (NT_50 _= 10, 95% CI, 7–13) (Figure 2e). The neutralizing titre of I-I-S against Omicron was 3.1-fold lower than that against Wuhan-1, and the neutralizing titre of I-I-I against Omicron was significantly lower than that against Wuhan-1 (9.9-fold) (Figure 2e).

### The titre of NAbs induced by both strategies decreased rapidly at 1M and 3M

**Figure 3.**
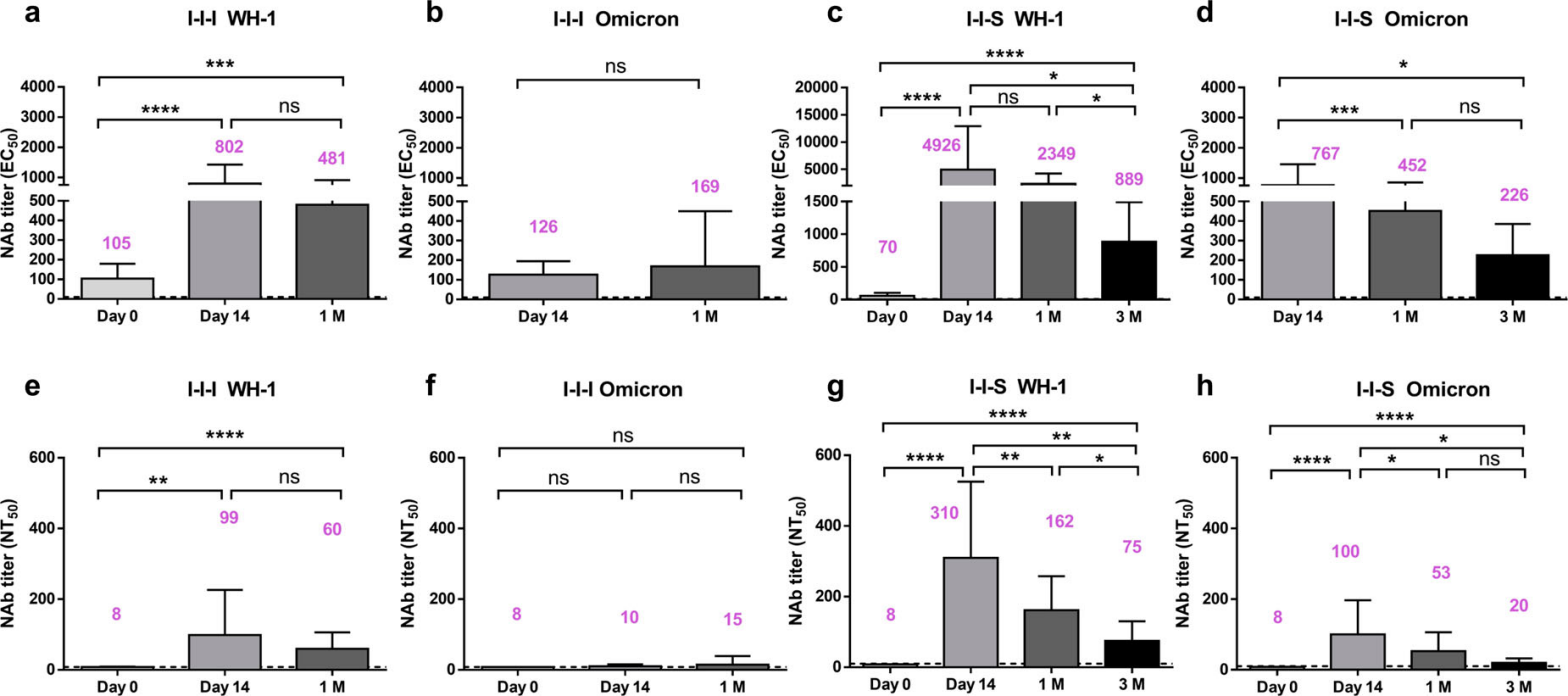
Duration of neutralizing antibody titres after booster vaccinations. (a, b) Long-term profile of EC_50_ titers based on pVNT against Wuhan-1 strain and Omicron strain for I-I-I group (n = 20 at D0 and D14, n = 13 at 1M). (c, d) Long-term profile of EC_50_ titres based on pVNT against Wuhan-1 strain and Omicron strain for I-I-S group (n = 25 at D0 and D14, n = 23 at 1M, n = 10 at 3M). (e, f) NT_50_ titres against Wuhan-1 strain and Omicron strain at D0, D14 and 1M post inactivated booster vaccination (n = 20 at D0 and D14, n = 13 at 1M). (g, h) NT_50_ titres against Wuhan-1 strain and Omicron strain at D0, D14, 1M and 3M post RBD subunit booster vaccination (n = 25 at D0 and D14, n = 23 at 1M, n = 10 at 3M). ns, not significant; *P < 0.05, ***P < 0.001, ****P < 0.0001.

For both boosters, the EC_50_, titre against the Wuhan-1 strain peaked at D14, which gradually declined, and there was no significant difference between the values at D14 and 1M (Figure 3a and c). However, the NAb titre at 3M (EC_50 _= 889, 95% CI, 453–1318) was 5.5-fold lower than that at D14 (EC_50 _= 4926, 95% CI, 1627–8225) after the third shot of the RBD recombinant subunit vaccine was administered (Figure 3c). In addition, in the I-I-I group, the EC_50_ titre against Omicron peaked at 1M (EC_50 _= 169, 95% CI, 0–339) post booster vaccination, which was not significantly higher than that at D14 (EC_50 _= 126, 95% CI, 94–159) (Figure 3b). In the I-I-S group, the EC_50_ titre against Omicron peaked at D14 (EC_50 _= 767, 95% CI, 482–1051), which decreased to 452 (95% CI, 279–625) and 226 (95% CI, 111–340) at 1M and 3M post booster vaccination, respectively (Figure 3d).

On one hand, our data showed that I-I-S induced high NAb titres against Wuhan-1 at EC_50_ 889 (95% CI, 453–1318) and against Omicron at EC_50_ 226 (95% CI, 111–340) at 3M post booster vaccination, which was considerably higher than those at 1M after two inactivated vaccinations, as shown in Figure 1d (Figure 3(c and d)). On the other hand, in the I-I-S group, compared to the titres against the Wuhan-1 strain on D14 after the booster dose, the NAb titre decreased to 48% and 18% at 1M and 3M after the booster dose, respectively, indicating that NAb titres decrease rapidly after boosting, which raises concerns about the suitability of NAbs for vaccination strategies.

Data from the conventional virus neutralization test showed that the neutralizing titres against the Wuhan-1 strain for both booster strategies peaked at D14, which were 1.7-fold and 1.9-fold higher than that at 1M for I-I-I and I-I-S, respectively (Figure 3(e and g)). However, a 4.1-fold lower neutralizing titre was observed at 3M (NT_50 _= 75, 95% CI, 35–114) than at D14 (NT_50 _= 310, 95% CI, 221–399) after the third shot of the RBD recombinant subunit vaccine (Figure 3 g). In addition, I-I-I led to the maintenance of a low NAb titre against the Omicron strain (NT_50 _= 15, 95% CI, 0–30) at 1M post vaccination, which was similar to the values obtained at D14 post booster vaccination (Figure 3f). For I-I-S, the neutralizing titre against Omicron peaked on D14 (NT_50 _= 100, 95% CI, 60–140), which decreased to 53 (95% CI, 29–76) and 20 (95% CI, 10–29) at 1M and 3M post booster vaccination, respectively (Figure 3 h).

Furthermore, we found that the rate of reduction (3M/1M) of total anti-S + N IgG, anti-RBD IgG, and ACE2 competition-NAb titres after two complete vaccinations (I-I) was significantly higher than that after I-I-S at 1M and 3M post vaccination, indicating that compared to two doses of inactivated vaccination, booster vaccination led to the maintenance of binding antibodies for a longer duration. In contrast, no significant difference was observed between I-I and I-I-S with respect to the rate of decline in NAbs against the Wuhan-1 and Omicron strains (Supplemental Figure 1), indicating that there was no difference in the decline in NAb titres between the I-I and I-I-S groups.

### I-I-S induced higher nAb titres against specific nAb clusters represented by the monoclonal antibodies 13g2 and 08b3

Two NAb clones, 13G2 and 08B3, were harvested from mouse hybridomas, which were prepared from SARS-CoV-2 RBD protein-immunized mice. 13G2 could neutralize the Wuhan-1 strain, but not the B.1.351 variant, whereas 08B3 could neutralize both strains (unpublished data). Our data showed that the NAb 13G2 and 08B3 titres declined more slowly than the total NAb titres within 12 months in recovered patients, and the titres of the two antibodies increased from 2 to 12 months after disease onset in some patients (unpublished data). The increase was considerably slower than that of anti-RBD and anti-N IgG titres, indicating that some neutralizing clones may be undergo affinity maturation for extended periods. Therefore, in this study, competitive ELISAs were performed to measure the levels of the two NAb clusters targeted or shared with the epitopes of the two NAb clones 13G2 and 08B3 in the I-I-I and I-I-S groups, to confirm potential differences in antibody maturation between the I-I-I and I-I-S groups.

**Figure 4.**
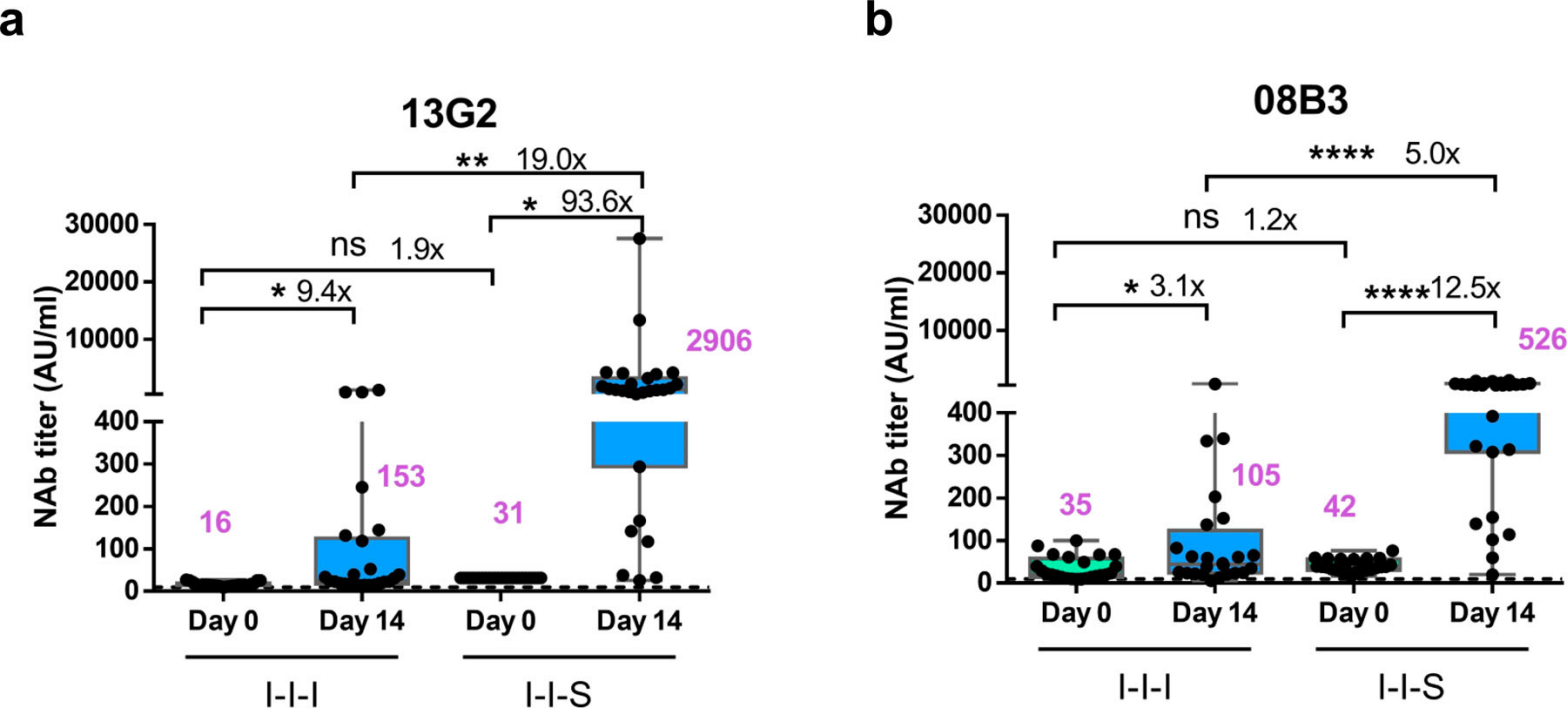
Neutralizing antibody titres against monoclonal antibodies 13G2 and 08B3 after booster vaccinations. (a) Comparison of neutralizing antibody titers based on monoclonal antibody 13G2 between I-I-I and I-I-S. n = 25 in I-I-I, n = 27 in I-I-S. (b) Neutralizing antibody titres against monoclonal antibody 08B3 for I-I-I and I-I-S group samples. n = 25 in I-I-I, n = 27 in I-I-S. ns, not significant; *P < 0.05, **P < 0.01, ****P < 0.0001.

The NAb titre against the clone 13G2 monoclonal antibody increased from 16 (95% CI, 14–18) to 153 (95% CI, 30–276) and from 31 (95% CI, 31–31) to 2906 (95% CI 690–5123) at D0 and D14 in the I-I-I and I-I-S groups, respectively (Figure 4a). Similarly, the NAb titre against the clone 08B3 monoclonal antibody increased from 34 (95% CI, 23–46) to 105 (95% CI, 40–170) and from 42 (95% CI, 36–48) to 526 (95% CI, 387–666) at D0 and D14 in the I-I-I and I-I-S groups, respectively (Figure 4b).

### I-I-S could target neutralizing epitopes more precisely than I-I-I

**Figure 5.**
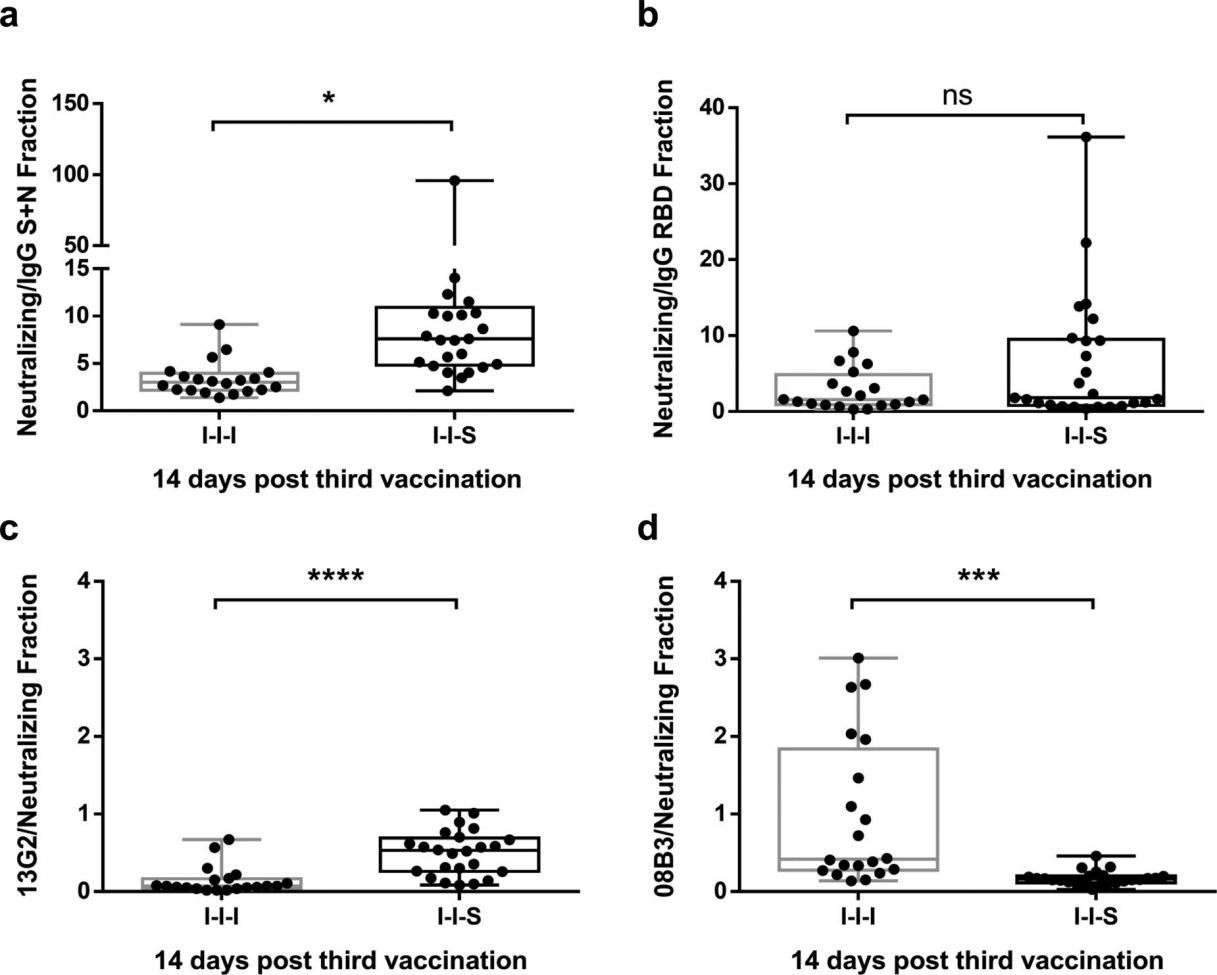
The portion of neutralizing epitopes targeted by booster vaccinations. (a) The portion of neutralizing antibody titre compared to total anti-S + N antibody for I-I-I and I-I-S groups. (b) The portion of neutralizing antibody titre compared to total anti-RBD antibody for I-I-I and I-I-S groups. The portion of neutralizing antibodies represented by monoclonal antibodies 13G2 (c) and 08B3 (d) compared to neutralizing antibody for I-I-I and I-I-S groups at D14 post booster vaccination. n = 20 in I-I-I, n = 25 in I-I-S. ns, not significant; *P < 0.05, ***P < 0.001, ****P < 0.0001.

To evaluate the quality of the immune response induced by booster vaccination, we analysed the fraction of NAb titres and total IgG antibodies. The results showed that the NAb titre and total anti-S + N or anti-RBD fractions in the I-I-S group were higher than those in the I-I-I group at 14 days post booster vaccination, which indicated that I-I-S targeted neutralizing epitopes more precisely (Figure 5a and b). In addition, we found that I-I-S targeted more 13G2 monoclonal antibody-representing epitopes but fewer 08B3 monoclonal antibody-representing epitopes than I-I-I (Figure 5c and d), indicating the significant difference in the composite patterns of NAbs.

### I-I-S induced a composite pattern of NAbs different from that induced by breakthrough infection

**Figure 6.**
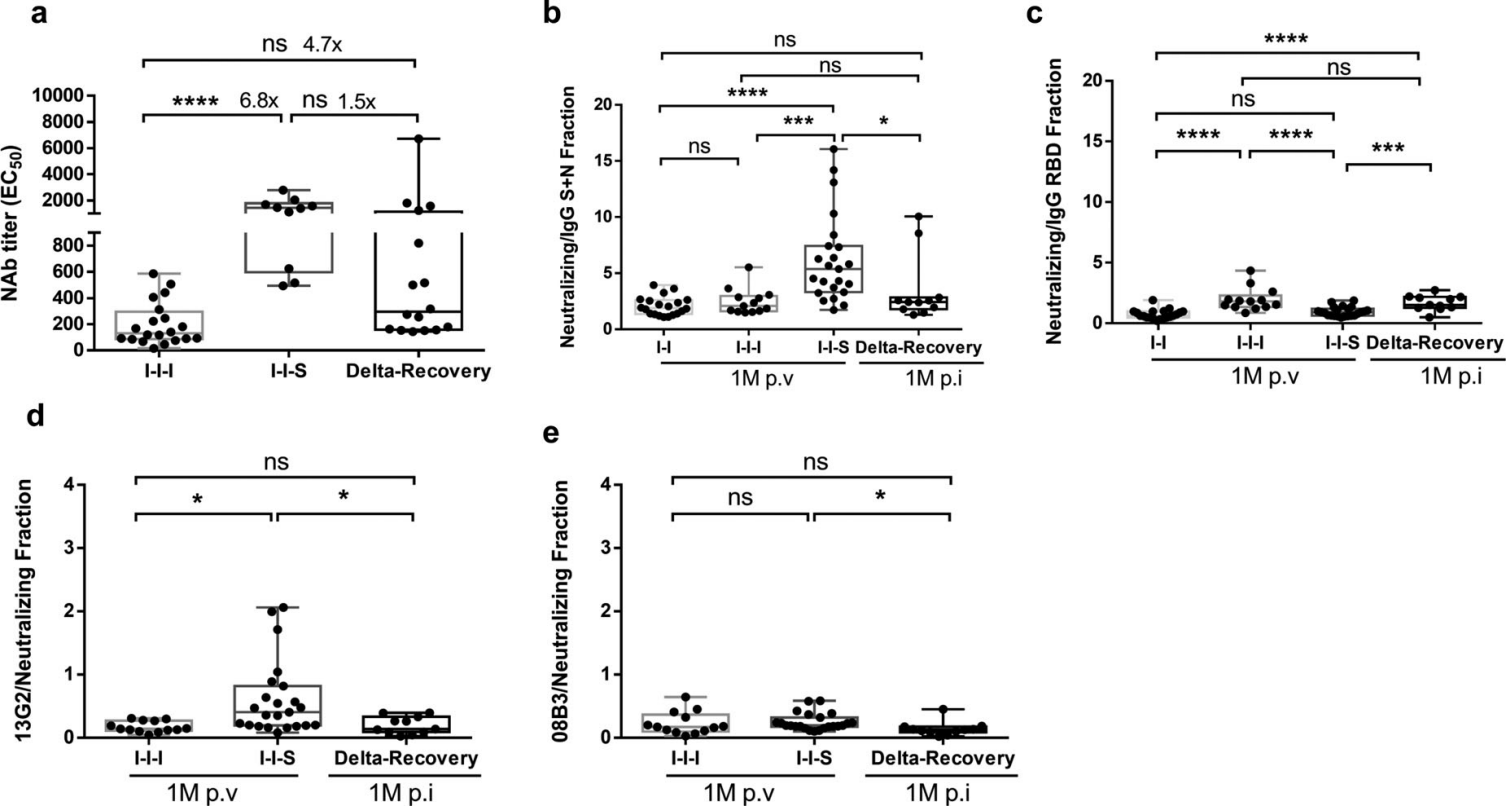
Different composite pattern between booster vaccination and breakthrough infection. (a) Neutralizing antibody titre against Wuhan-1 stain for samples collected at D14 post booster vaccinations and D14 post breakthrough infection. n = 20 in I-I-I, n = 10 in I-I-S, n = 16 in recovered Delta breakthrough patients. (b**,** c) The portion of neutralizing antibody titre compared to total anti-S + N and anti-RBD antibody at 1M post two-dose vaccination, post booster vaccinations and post breakthrough infection. n = 20 in I-I, n = 13 in I-I-I, n = 23 in I-I-S, n = 11 in Delta-Recovery. (d**,** e) The portion of neutralizing antibodies represented by monoclonal antibody 13G2 or 08B3 compared to total neutralizing antibody for vaccination samples and breakthrough infection samples. n = 13 in I-I-I, n = 23 in I-I-S, n = 11 in Delta-Recovery. ns, not significant; *P < 0.05, ***P < 0.001, ****P < 0.0001.

Here, we compared the level of protective immunity induced by I-I-S, I-I-I, and natural breakthrough infections (Figure 6), because natural infection generally confers better protective immunity than vaccination, not only in magnitude but also with respect to the immune components and quality. Patients with breakthrough infection were the ones who had received two doses of inactivated vaccines but were infected with the Delta strain. Samples were collected from recovered patients with breakthrough infection at D14 and 1M after disease onset. At D14 post vaccination/infection, compared to natural infection, I-I-S induced a marginally higher NAb titre against the Wuhan-1 strain (Figure 6a).

As antibody inclusion relationships, such as NAbs in total binding antibodies (anti-RBD and anti-N + S) and monoclonal NAbs in total NAbs, can reflect the antibody composite patterns, we compared the abovementioned fractions. We found that compared to breakthrough infection, I-I-S significantly increased the fraction of NAbs in total anti-S + N antibodies (Figure 6B) and the fractions of 13G2 (Figure 6D) and 08B3 (Figure 6E) in total NAbs, but reduced the fraction of NAbs in total anti-RBD antibodies (Figure 6C), at 1M post booster vaccination/infection. This indicated that I-I-S induces a different protective antibody pattern from breakthrough infection and is more specific for some neutralizing epitopes.

### I-I-S enhanced virus-specific CD4 + responses more potently than I-I-I

**Figure 7.**
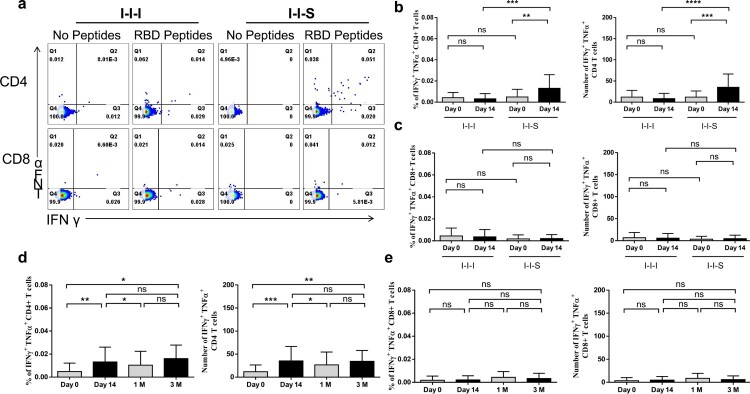
Cell responses after booster vaccinations. (a) Representative dot plots showing IFN-γ and TFN-α expression in CD4 + and CD8+ T cells after peptides stimulation at D14 post booster vaccination. (b) The percentages (left panel) and number/million PBMC (right panel) of IFN-γ+TFN-α+ CD4+ T cells after booster vaccination. (c) The percentages (left panel) and number/million PBMC (right panel) of IFN-γ+TFN-α+ CD8+ T cells after booster vaccination. n = 24 in I-I-I, n = 27 in I-I-S. IFN-γ+TFN-α+ CD4+ T cell response (d) and IFN-γ+TFN-α+ CD8+ T cell response at different time points post RBD recombinant subunit booster vaccination (e). n = 27 at D0 and D14, n = 23 at 1M, n = 10 at 3M. ns, not significant; *P < 0.05, **P < 0.01, ***P < 0.001, ****P < 0.0001.

Analysis of the immune response after booster vaccination showed that compared to that at D0, a significant increase in the RBD-specific CD4+ T cell population was observed in the I-I-S group at D14 (Figure 7a) (12 (95% Cl, 6–18) to 35 (95% Cl, 23–48) per million PBMCs from D0 to D14), which was significantly higher than that observed in the I-I-I group (Figure 7b). However, no obvious increase in the virus-specific CD8+ T cell population was observed after either booster was administered (Figure 7c). Furthermore, to evaluate the maintenance of the T cell response after RBD subunit booster vaccination, PBMCs collected at different time points post vaccination were stimulated with RBD peptide pools, and the frequency of IFN-γ- and TFN-α-expressing T cells was measured. At 3M post booster vaccination, 34 (95% Cl, 18–51) IFN-γ+ and TFN-α+ CD4+ T cells were observed, which was significantly higher than the frequency at D0, but did not differ from those at D14 and 1M (Figure 7d). However, the frequency of virus-specific CD8+ T cells remained low at 3M post booster vaccination (Figure 7e).

## Discussion

Although COVID-19 vaccines continue to provide protection against severe disease, hospitalization, and death, thus far, neither the immune correlation with protection nor the duration of protection has been established for COVID-19 vaccines. Studies have suggested a correlation between the efficacy/effectiveness of different vaccines against symptomatic diseases and mean NAb titres. However, vaccine-induced immunity wanes over time, and the decline in the degree of immunity may differ for different vaccines, target populations, circulating SARS-CoV-2 strains (VOCs in particular), and intensities of exposure [14,15]. In this study, after two completely inactivated vaccines had been administered, the anti-RBD IgG, anti-N + S IgG, and ACE2 competing NAb titres peaked at approximately 1M but declined at 3M, waning to lower levels at 6M and becoming undetectable in some individuals, which is consistent with the findings of most observational studies on vaccines, such as mRNA vaccines [16,17].

In the long term, it is important to boost protective immunity components (such as NAbs) to high levels. However, the recommendation of a booster dose requires a complex evaluation that considers the global vaccine strategy beyond clinical and epidemiological data. Several clinical trials in the US found that the boosters of all three COVID-19 vaccines (Moderna, Pfizer, and Johnson adenovirus vaccines) increased NAb titres, irrespective of the type of booster or primary vaccine series used [18]. Currently, in China, the vaccines available are limited to inactivated vaccines, adenoviral vector vaccines, and recombinant subunit protein vaccines, and booster vaccines should be selected carefully and optimized. The administration of two inactivated vaccines followed by an inhaled adenoviral vector COVID-19 vaccine reportedly induced higher NAb titres and stronger virus-specific T cell responses than those elicited by three shots of inactivated vaccines [19]. Meanwhile, two recent studies reported that a third dose of RBD recombinant subunit vaccine significantly boosted NAb titres and also enhanced the neutralizing capacity against five VOCs, including the Delta variant; however, data on the Omicron strain are missing as the strain had not emerged when the study was conducted [20,21]. Because Omicron variants have been detected in more than 100 countries, they may contribute to more than 75% of the recently confirmed cases in the USA (nearly 1,000,000 per day), and Omicron may become the dominant pandemic strain in the near future. As independent data, the findings of our study confirmed that inactivated-subunit vaccines are highly effective as a prime-boost strategy. More importantly, our data showed that I-I-S induced a considerably high NAb titre against Omicron at D14 and 1M post booster vaccination, which was similar to the NAb titre induced by the third shot of the Pfizer mRNA vaccine [22]. Additionally, we demonstrated the high immunogenicity of I-I-S with respect to not only the total NAb titre but also individual NAb clusters. I-I-S could induce five times more NAbs than I-I-I, suggesting that the introduction of a relatively large protein titre into the body can induce a robust recall of antibody responses. However, our data showed that NAb titres decline rapidly. The EC_50_ titre against the Wuhan-1 strain decreased by half within 1 month in the I-I-I group and to 48% and 18% of that at D14 by 1M and 3M after RBD subunit boosting, and the NT_50_ titre decreased to 52% and 24% of that at D14 by 1M and 3M after the third shot of the RBD subunit vaccine. Similarly, the NT_50_ against Omicron also decreased to 53% and 20% of that at D14 by 1M and 3M post RBD subunit booster vaccination, indicating that subunit vaccine-boosted immunity is unsuitable for maintaining antibody titres in the long term; therefore, in future, COVID-19 vaccines may require boosting annually or more frequently, especially under the emergence of new immune-evading mutant variants. Furthermore, since most amino acid mutations in different variants primarily occur in the ACE2-binding site (RBD domain) of the spike protein, and most other domains remain well conserved, it is possible that T cell immunity can help combat different VOCs. Importantly, our data demonstrated that I-I-S induced stronger viral CD4+ T cell responses than I-I-I or two inactivated complete vaccinations. Therefore, based on T cell immunity, heterologous boosting with the RBD subunit will provide broader protection than I-I-I and two-dose vaccinations.

The emergence of SARS-CoV-2 variants has raised concerns regarding the range of NAb responses. Vaccination programs and the increasing numbers of natural infections continue to strengthen herd immunity; conversely, this exerts selection pressure on SARS-CoV-2 to develop additional immune escape mutations. Therefore, not only the titre but also the quality and range of neutralizing capacity of vaccine-induced NAbs should increase. Natural infection can enhance the range of neutralizing effects against SARS-CoV-2 at 1 year after infection [23], and B cell clones expressing broad and potent antibodies are selectively retained in the repertoire over time and expand markedly after vaccination. Our data showed that I-I-S could induce the production of a higher proportion of NAbs in total RBD IgG antibodies than I-I-I, indicating that the subunit booster can target more neutralizing epitopes precisely than whole inactivated virus particles. Moreover, compared to that in patients who developed and recovered from breakthrough infection with the Delta variant after receiving two doses of inactivated vaccine, I-I-S induced marginally higher levels of total NAbs. However, at the individual level, the titres of two monoclonal represented clones were significantly higher than those in patients who recovered from Delta infection, indicating that I-I-S could boost certain advantageous epitopes more effectively than natural infection. Therefore, it will be interesting to investigate different vaccine strategies with respect to somatic mutations in antibodies, memory B cell clonal turnover, and monoclonal antibody development.

Overall, this small-scale study have demonstrated a useful boost strategy based on inactivated and recombinant subunit vaccines. In view of the emergence of immune escape variants, our strategy could induce higher levels of NAbs with a broad neutralizing capacity against different variants, although further investigation is needed to achieve long-term immunity.

## Supplementary Material

Supplemental Material
