# Supplementary material for: Heterologous boosting with third dose of coronavirus disease recombinant subunit vaccine increases neutralizing antibodies and T cell immunity against different severe acute respiratory syndrome coronavirus 2 variants
Source: Emerg Microbes Infect. 2022 Mar 15;11(1):829–40. doi: 10.1080/22221751.2022.2048969 (PMC8928863; doi:10.1080/22221751.2022.2048969)
Supplement: Supplemental Material [file TEMI_A_2048969_SM4163.docx]

# Supplementary information

In order to compare the maintenance of antibody response between two shots of inactivated vaccination and third shot of recombinant RBD subunit vaccination, we analyzed the rate of reduction (3M/1M) of anti-A+N IgG, anti-RBD IgG and ACE2 competition neutralizing after after two complete vaccination (I-I) and I-I-S. We found that the rate of reduction (3M /1M) of total anti-S+N IgG, anti-RBD IgG and ACE2 competition neutralizing antibodies of I-I was significantly higher than that of I-I-S at 1M and 3M post vaccination, indicating that booster vaccination induced longer maintaining of those binding antibodies than only two dose of inactivated vaccination. In contrast, no significant difference was observed between I-I and I-I-S as concerns as the rate of reduction for neutralizing antibody against Wuhan-1 and Omicron (Supplementary figure 1), indicating there is no difference in the decay of neutralizing antibodies between I-I and I-I-S.


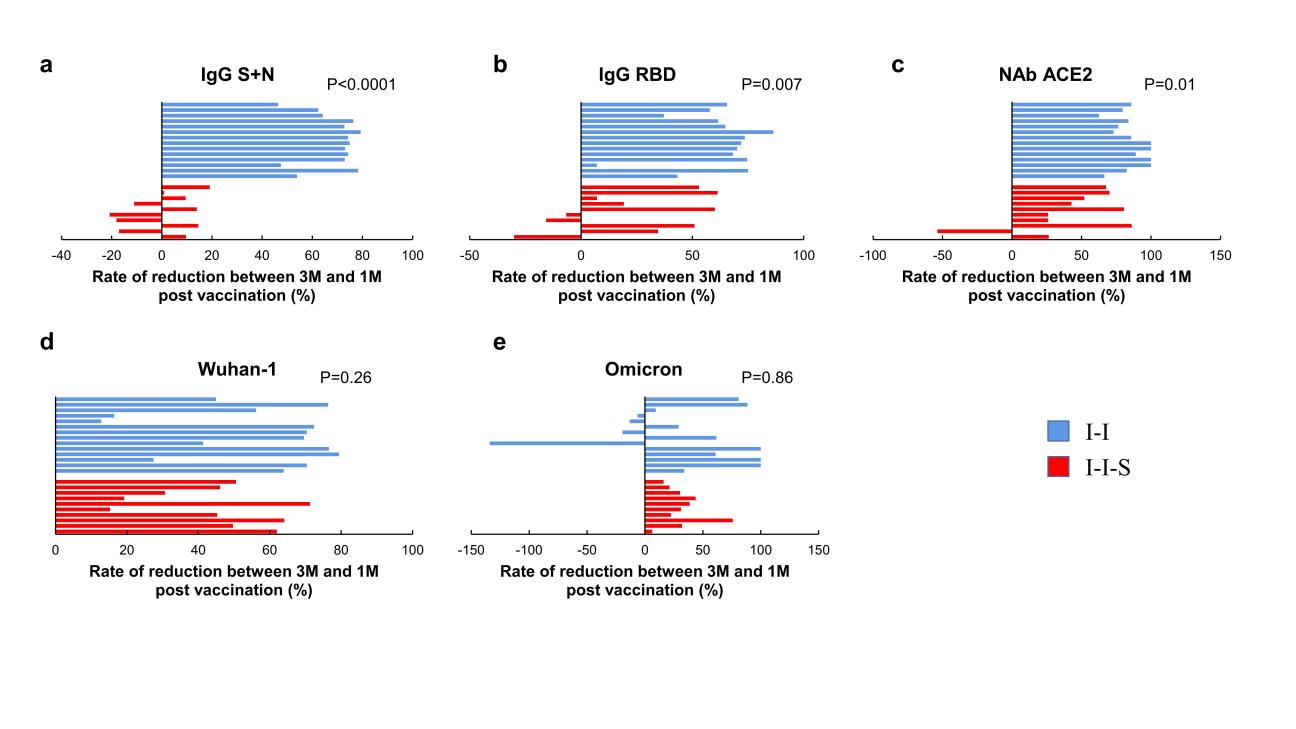


**Supplementary Figure 1.** The rate of reduction between 3M and 1M post vaccination. (a) The reduction rate between 3M and 1M of anti-S+N IgG for samples collected after two-dose of inactivated vaccine and third shot of RBD subunit vaccine. (b) The reduction rate of anti-RBD IgG for I-I and I-I-S. (c) The reduction rate of ACE2 completing neutralizing antibody titre for I-I and I-I-S. (d, e) The reduction rate of neutralizing antibody against Wuhan-1 and Omicron strain for I-I and I-I-S.
